# Exploring Compersion: A Study on Polish Consensually Non-Monogamous Individuals and Adaptation of the COMPERSe Questionnaire

**DOI:** 10.1007/s10508-024-02930-5

**Published:** 2024-07-01

**Authors:** Klara Austeja Buczel, Paulina D. Szyszka, Izu Mara

**Affiliations:** 1 Doctoral School in the Social Sciences, Jagiellonian University, 34 Rynek Główny, 31-010 Kraków, Poland; 2 Institute of Psychology, Jagiellonian University, Kraków, Poland; 3 Psychology Department, SWPS University of Social Sciences and Humanities, Katowice, Poland

**Keywords:** Compersion, Consensual non-monogamy, Relationships, Polyamory

## Abstract

Compersion is a positive emotion experienced in relation to one’s partner’s relationship(s) with other partner(s). Experiencing it is highly desired in communities practicing consensual non-monogamy (CNM), especially polyamory. This article presents the results of a study on compersion on Polish CNM individuals. The main goal of the study was to adapt to the Polish-speaking population the COMPERSe (Classifying Our Metamour/Partner Emotional Response Scale; Flicker et al., 2021), the first standardized quantitative scale designed to measure compersion. The analyses were performed on data obtained from 211 individuals in CNM relationships and on a comparative group of 169 people in monogamous relationships. The results of the confirmatory factor analyses suggested that the three-factor model of the original COMPERSe version did not fit well, leading to further revisions that resulted in a 7-item, two-factor solution with excellent fit, excellent internal consistency, strong divergent and convergent validity, and excellent test–retest stability. The CNM individuals were found to have higher scores on compersion and cognitive empathy and were also less jealous than the monogamous participants. Furthermore, polyamorous individuals experienced more compersion and less aversion to partner’s autonomy than people in open relationships. It was also revealed that compersion indirectly predicted relationship satisfaction by decreasing jealousy and that compersion was, in turn, predicted by cognitive empathy. However, when polyamorous and open relationships were analyzed separately, compersion predicted relationship satisfaction directly, but only in polyamorous relationships; meanwhile, in open relationships, satisfaction was directly predicted by cognitive empathy.

## Introduction

In Western society, monogamy is one of the most strongly held norms (Conley et al., 2013). Yet, there is a rising amount of people who break that norm by participating in consensual non-monogamy (CNM), sparking interest in researchers all around the globe (Barker & Landridge, 2010). The fact that one’s experience of their partner loving other person can bring someone joy may seem unusual, even for mental health practitioners (Grunt-Mejer & Łyś, 2022), and is something that researchers have been exploring in more detail for less than 20 years (e.g., Duma, 2009; Ritchie & Baker, 2006).

Compersion, the feeling mentioned, can be loosely defined as a pleasant, joyful, and satisfying feeling that appears when one is aware of (either by observing or knowing) the sexual and/or romantic relationship of one’s partner with someone else (Mogilski et al., 2019). It is mostly connected to polyamory, which refers to engagement in multiple intimate, sexual, and/or romantic relationships (e.g., Haritaworn et al., 2006), contrary to the open relationships, which are often referred to as primary couples who engage in sexual (but typically not romantic) relationships with people outside of the couple (e.g., Conley et al., 2017; cf. Fairbrother et al., 2019; see, however, for controversies regarding the definition of an open relationship: Cohen, 2016). As reported in the study by Flicker et al. (2021), some people consider it the opposite of negatively valenced emotions, such as jealousy, while others emphasize joy, excitement, contentment or love, and pride/validation. Although the study by Flicker et al. (2021) did not test causal relations between compersion and other constructs, the authors proposed three mechanisms by which people may feel compersion: an empathy-like mechanism, an emotion contagion, and a positive benefit for oneself. However, it is worth mentioning that the term itself can be defined in various ways as different people put emphasis on its different aspects (e.g., Brunning, 2020; Flicker et al., 2021).

International research on compersion is only now gaining momentum. Up until recently (e.g., Balzarini et al., 2021; Flicker et al., 2021; Hunter & Stockwell, 2022), there were only a few articles concerning it in more detail, oftentimes not using standardized methodological measures (especially in quantitative studies; see, e.g., Aumer et al., 2014; Duma, 2009).

CNM and related phenomena are even more niche topics in Polish research. Although the term “compersion” is used by the CNM community and researchers outside Poland for at least 20 years (e.g., Anapol, 1998; Ritchie & Barker, 2006) with one study dating back as far as 43 years (Pines & Aronson, 1981), the Polish version of the word can only be tracked back to 2012 (Andrzejczyk-Bruno, 2012), followed by a chapter by Grunt-Mejer (2015). When searching “kompersja” (Polish for compersion) or looking for studies on compersion performed on the Polish population, Google Scholar shows fewer than 10 results (search date: March 19, 2023). Most of them are unpublished dissertations (Frycz, 2019; Jagiełło, 2022; Michalczak, 2014; Pokładowska, 2015). Only one study (Frycz, 2019) used a quantitative procedure in which compersion was measured using an unvalidated questionnaire (Duma, 2009). Thus, in Poland, there is certainly a demand for a new line of research on CNM, as well as a validated and reliable tool for measuring compersion.

The COMPERSe (Flicker et al., 2021), compared to other ways of measuring compersion used by researchers (e.g., the unvalidated questionnaire by Duma [2009] or modified jealousy measurements [Mogilski et al., 2019]), seems to provide the most reliable information on compersion level in an individual, as it is the only standardized tool for measuring compersion to date. It has a three-factor structure, possibly mapping differences in types of compersion or areas of its occurrence: (1) sexual arousal to one’s partner relationship with metamour (Sexual Arousal), (2) joy and contentment of one’s partner relationship with metamour (Happiness about Partner/Metamour Relationship), and (3) excitement and thrill about the perspective of a new intimate relationship or sexual encounter of one’s partner (Excitement for New Connections). As there are almost no Polish studies on compersion, we hope to facilitate future research by providing Polish professionals with a validated tool.

The aim of this article is to validate the Polish version of the COMPERSe (Flicker et al., 2021). We describe the steps involved in the scale adaptation, including confirmatory factor analysis, establishing reliability, test–retest stability, and convergent and divergent validity. For this study main goal, we tried to follow recommendations and guidelines for the best practices in scale adaptation (e.g., Cicchetti, 1994; Worthington & Whittaker, 2006), regarding all steps mentioned in previous sentence, including number of participants per parameter for confirmatory factor analysis, cutoff values for fit indices (Hu & Bentler, 1999; Jöreskog & Sörbom, 1982), methods to estimate reliability (Cronbach, 1943; Yen & Lo, 2002), and methods to establish different types of validity (Carmines & Zeller, 1979). Aside from scale adaptation, we also want to address some of the issues mentioned by Flicker et al. (2021) in their discussion, such as potential differences in experiencing compersion in various forms of CNM and exploring factors that facilitate compersion.

### Pilot Study

In our first step, we conducted a pilot study whose purpose was to establish the nomenclature of the term *metamour*, which has no equivalent in the Polish language. It was important as most of the COMPERSe items contain this term. Metamour is a label used in CNM (especially polyamorous) communities that describes a person who is a partner of one’s partner but is not romantically or sexually related to the one. That said, it is a person for whom compersion is felt.

We posted a survey on a Facebook group associating Polish people in CNM relationships, in which we asked what translation of the metamour do they use or prefer. The survey was structured in such a way as to allow people to add their own options, which could also be voted on by the general public. There was the possibility to vote for more than one option.

Seventy-five people participated in the pilot survey. A total of 61% found that the option *metamour* (version without direct translation) was the best. A total of 24% chose the term *kochanek*, which can be translated into English as “lover.” However, this option was somewhat controversial because many people drew attention to the fact that this term has some negative association with cheating. The third option most voted for (20%) was *metapartner* and other options were chosen by less than 10% of the participants. Public comments to the survey mainly indicated a preference for no direct translation (*metamour*) or *kochanek*. Taking into account all votes, we decided to use the untranslated *metamour* option.

### Main Study

The purpose of the main study was to adapt the COMPERSe to the Polish-speaking population. The study was preregistered. Our first step was to translate and back-translate the questionnaire and then test the scale on the CNM sample. The next steps were to analyze whether the three-factor structure fits the data and then establish reliability, test–retest stability, and convergent and divergent validity of the questionnaire. Aside from the adaptation, we also wanted to examine the differences between experiencing compersion among non-monogamous and monogamous participants, as well as differences in compersion between different kinds of CNM.

### Divergent and Convergent Validity

To establish divergent and convergent validity, we selected measures based on Flicker et al. (2021) study. When possible, we used the same measurement tools as in the original research; when they were not available in the Polish version, we did our best to find equivalents. Additionally, we decided to test several new hypotheses regarding relationship of compersion with other variables; the rationale for each hypothesis is written in relevant paragraph below. Apart from the analyses performed on CNM participants, we were also interested in exploring relationships between compersion and other selected variables in monogamous sample, but we did not make any predictions about the direction of these relationships beforehand.

First, we expected the compersion to be negatively related to jealousy. Some qualitative research showed that some people perceive jealousy as the opposite of compersion (Flicker et al., 2021) or suggest that compersion is perceived as a counterpart to jealousy or can mitigate feelings of jealousy (Deri, 2015; Rubinsky, 2018). In some quantitative research, negative relations between jealousy and compersion have been shown (Aumer et al., 2014; Duma, 2009; Flicker et al., 2021).

The second major correlate of compersion in the study by Flicker et al. (2021) was empathy. In the qualitative part of the study, participants mentioned that empathy is the same or is a driving factor of compersion, which was reflected in the quantitative analysis, where both emotional and cognitive empathy were positively related to compersion. Thus, we expected similar results in our study. Following other results from the original research, we also expected compersion to be positively related to the tendency to experience positive emotions and higher relationship satisfaction.

In addition to previously established correlates, we also assumed that compersion would be negatively related to anxiety, especially as a trait. Although the original study by Flicker et al. (2021) did not demonstrate any relation between compersion and the tendency to experience negative emotions (measured by the Big Five personality trait of Neuroticism), the unpublished Polish study indicated that compersion was negatively related to anxiety among polyamorous people (Frycz, 2019). Thus, we expected similar results in our research.

We also expected compersion to be positively related to emotional intelligence (EI). According to the EI ability model (Mayer et al., 2000, 2016), emotional intelligence can be defined as one’s competencies related to perception, understanding, and managing emotions, as well as the integration of these processes with cognition to promote personal growth. Therefore, high EI allows for efficient use of emotions for interpersonal or intrapersonal use. Since negative feelings (spec. jealousy) are generally communicated in CNM relationships to see affirmation and validation (Rubinsky, 2018), it seems reasonable to expect people with higher EI to better manage their emotional states, consequently showing a greater tendency to experience compersion.

High self-esteem is associated with building more satisfying relationships (Luciano & Orth, 2017). In the case of CNM and compersion, it is possible that high self-esteem may have possible protective function similar to compersion: Since in CNM relationships comparisons with others (especially with metamours) are feasible and vivid, there are many moments when one may feel more insecure due to perceive oneself as less valuable to one’s partner than one’s metamour(s). High self-esteem may help one deal with the negative effects of these comparisons (cf. Ben-Ze’ev, 2022), thus preventing creation of negative attitudes toward metamour(s) and potentially promoting more positive attitudes, i.e., compersion. Also, practicing polyamory can improve self-esteem (Wolfe, 2003). Thus, we expected a positive correlation with self-esteem and compersion. Also, as self-esteem is not a one-dimensional phenomenon and can be divided into feelings of social worth and feelings of efficacy and control (Tafarodi & Swann, 2001), we also expected to observe differences between these two dimensions of self-esteem. Namely, we speculated that it is more likely that compersion would be more related to feelings of social worth rather than feelings of efficacy and control, since it is the first which is more related to social interactions and thus to complex CNM relationships with much more possible comparisons with others.

Finally, several studies have mentioned the relationship between attachment styles and CNM. A study by Ka et al. (2022) suggested that secure attachment style strengthens the relationship between sociosexual behavior and attitudes toward CNM and that anxious attachment does not moderate the relationship between sociosexual behavior and willingness to engage in CNM. Moors et al. (2015) reported that among people without experience in CNM, avoidant individuals had more positive attitudes and were more willing to hypothetically engage in CNM (see also Feeney & Noller, 1990), and that highly anxious people were more negative toward CNM, but the anxious style was not related to the desire to engage in CNM. Additionally, one Polish study indicated that polyamorous individuals were more securely attached than monogamous people (Frycz, 2019). Nevertheless, these results do not indicate any relation between actual feelings of compersion and attachment styles. However, some findings indicated that CNM people report relationship benefits similar to those reported by secure individuals (Barker, 2005; Bonello & Cross, 2010; de Visser & McDonald, 2007). Other researchers (Balzarini et al., 2017, 2019) found that people engaged in CNM report high-quality relationships, open communication, honesty, trust, intimacy, and less jealousy, qualities linked to a secure attachment style (Feeney, 2008). These qualities may potentially influence how one perceives one’s “potential threats” to one’s relationship–feeling more secure with one’s current partner and not being in fear of either abandonment or neglect of one’s needs may be an important prerequisite of more positive feelings toward these “potential threats,” i.e., metamours, as they may be not perceived as “threats” anymore. Furthermore, open communication of one’s needs and insecurities may improve CNM relationship’s quality, subsequently translating into an increase in the potential for feeling compersion. Therefore, we expected compersion to be positively related to secure attachment and negatively related to anxious and avoidant attachment styles.

In sum, we hypothesized that the COMPERSe scores would be positively related to (1) emotional and cognitive empathy toward one’s partner, (2) the tendency to experience positive affect, (3) emotional intelligence, (4) self-esteem (overall and self-liking dimension, not necessarily self-competence), (5) relationship satisfaction, and (6) secure attachment style. Furthermore, we expected the COMPERSe to be negatively related to (1) jealousy, (2) trait anxiety, and (3) anxious and avoidant attachment styles.

### Additional Hypotheses

In addition to the adaptation of the COMPERSe, we wanted to test several other hypotheses, especially on the differences between different types of relationships. Previous research indicated that monogamous people tend to be more distressed in reaction to scenarios of emotional and sexual extra-pair involvement of their partner compared to CNM people (Mogilski et al., 2019). Furthermore, CNM individuals reported lower overall and sexual jealousy (Valentova et al., 2020), and people in polyamorous relationships reported less jealousy and more compersion with their partners (Balzarini et al., 2021). Therefore, we expected to observe a higher level of compersion among CNM individuals compared to monogamous individuals and lower levels of jealousy among CNM individuals compared to monogamous individuals. Also, we expected some differences between people involved in different types of CNM relationships. As we mentioned in the Introduction section, polyamory—but not open relationships—is the type of relationship usually associated with maintaining engagement in multiple intimate, sexual, and/or romantic relationships (e.g., Haritaworn et al., 2006). Given that polyamory is the only major type of CNM relationship not assuming emotional exclusivity, it is possible that individuals in polyamorous relationships would have higher levels of the emotional dimension of compersion, at the same time not differing in terms of sexual dimension levels compared to people in an open or a “friends with benefits” (FWB) type of relationships.

Furthermore, we were also interested in examining whether higher compersion is related to higher relationship satisfaction and, if so, whether this relationship is direct or indirect. Previous research indicated inconsistent results: On the one hand, in Aumer et al. (2014) study, compersion was related to relationship satisfaction only for women who were single or in open relationships but was unrelated for monogamous or polyamorous women or for men in general. On the other hand, Flicker et al. (2021) showed a positive correlation between relationship satisfaction and compersion, and Balzarini et al. (2021) reported that anticipated compersion was associated with greater relationship satisfaction. These inconsistencies may be because Aumer et al. (2014) used the unstandardized measurement tool, and it is possible that with the use of the COMPERSe we will show a positive relationship between compersion and relationship satisfaction for CNM individuals overall.

We also wanted to examine the role of possible facilitators of compersion, especially to determine whether cognitive empathy would predict the level of compersion, as it was mentioned as a possible predictor of compersion throughout several studies (cf. Flicker et al., 2021, 2022).

## Method

### Participants

The minimal sample size required to conduct CFA was determined using the A-priori Sample Size Calculator for Structural Equation Models (Soper, 2021). Given 11 observed and three latent variables, it was indicated that a minimum of 123 participants would be sufficient, considering the structure of the model and the detection of a medium-sized effect (0.30) with power = 0.80.

Participants were recruited online from social media groups associating Polish people in CNM relationships and through snowball sampling. For monogamous sample, participants were recruited from thematic social media groups regarding close relationships that did not contained references to non-monogamy. Apart from the description of the research information approved by the Research Ethics Committee, participants were also presented with an information regarding definition of consensual relationship—a relationship in which every person involved agree to all the rules established for that relationship, referring mainly to the exclusivity, i.e., to the number of people involved in an intimate relationship with each person in the relationship. (This was applied for both non-monogamous and monogamous types of relationships, as we did not wanted to include cheating individuals.) A total of 749 people accessed the study, including 247 who did not complete the COMPERSe and were excluded from the study. The remaining sample of 502 individuals was screened according to inclusion criteria analogous to those used by Flicker et al. (2021): Polish-speaking, at least 18 years old and currently in at least one intimate relationship. A total of 68 participants did not meet these inclusion criteria and were excluded from the sample. The sample was then extracted from 169 people who reported being consensually monogamous. From the remaining CNM sample, we excluded 54 participants who reported not having any partner who also had at least one current intimate partner (i.e., metamour).

The final CNM sample consisted of 211 participants (age range 18–51), and the monogamous sample (which we will refer to as CM = consensually monogamous) consisted of 169 participants (age range 18–53), which was sufficient for the required analysis. Detailed information on demographics and relationship characteristics is shown in Table 1.

**Table 1:** Participant demographics and relationship characteristics for the consensually non-monogamous and consensually monogamous subsamples

Characteristic	CNM sample (*n* = 211)		CM sample (*n* = 169)	
	M/n	SD/%	M/n	SD/%
Age (years)	28.40	7.22	28.25	7.15
*Gender of the participant*^a^				
Agender	4	1.90	–	–
Demiboy	–	–	1	0.59
Demigirl	2	0.95	2	1.18
Genderfluid	5	2.37	2	1.18
Genderqueer	3	1.42	1	0.59
Man	50	23.70	37	21.89
Non-binary	42	19.91	15	8.88
Woman	107	50.71	111	65.68
Unspecified/something else/non-labeled	4	1.90	1	0.59
*Sexual orientation*^a^				
Asexual	8	3.79	2	1.18
Aromantic	1	0.47	–	–
Bicurious	4	1.90	2	1.18
Biromantic	1	0.47	–	–
Bisexual	86	40.76	38	22.49
Demiromantic	–	–	1	0.59
Demisexual	11	5.21	6	3.55
Enbian	2	0.95	–	–
Finromantic/gynromantic^b^	1	0.47	–	–
Finsexual/gynsexual^b^	6	2.84	2	1.18
Heteroflexible	10	4.74	3	1.78
Heteroromantic	2	0.95	1	0.59
Heterosexual	45	21.33	85	50.30
Homoflexible	3	1.42	–	–
Homoromantic	1	0.47	1	0.59
Homosexual	11	5.21	18	10.65
Minsexual/androsexual^b^	4	1.90	5	2.96
Omnisexual	–	–	1	0.59
Panromantic	–	–	2	1.18
Pansexual	48	22.75	17	10.06
Polysexual	1	0.47	1	0.59
Queer	6	2.84	4	2.37
Questioning	1	0.47	–	–
Unspecified/something else/non-labeled	7	3.32	3	1.78
*Participant’s approach to relationships*^a^				
Friends with benefits	62	29.38	–	–
Monogamy	–	–	169	100.00
Open relationship	102	48.34	–	–
Polyamory	130	61.61	–	–
Queerplatonic	2	0.95	–	–
Relationship Anarchy	4	1.90	–	–
Swinging	18	8.53	–	–
Unspecified/something else/non-labeled	12	5.69	–	–
*Type of relationship with partner*				
Friends with benefits	24	11.37	–	–
Monogamy	–	–	169	100.00
Open relationship	70	33.18	–	–
Polyamory	99	46.92	–	–
Queerplatonic	2	0.95	–	–
Relationship Anarchy	1	0.47	–	–
Swinging	9	4.27	–	–
Unspecified/something else/non-labeled	6	2.84	–	–

^a^Percentages may exceed 100% because participants are able to choose more than one option

^b^Finsexual, Minsexual—we chose to use these terms to describe individuals who are attracted to people who are Feminine in Nature (FIN) or Masculine in Nature (MIN) in either their gender and/or presence. The attraction may be extended to any feminine/masculine-aligned genders (LGBTQIA+ Wiki, 2022). Although there are also terms that can be used synonymously, such as gynesexual and androsexual (cf. Brito, 2019), some people sometimes regard them as somewhat transphobic by reducing individuals to their genitals (Tame One, 2018). Furthermore, the terms finsexual and minsexual were directly used in our research by four people. Thus, we assigned them and anyone who defined their preferences as “to all people who are feminine/masculine” to these categories

## Measures

### Demographic and Relationship Questions

At the beginning of the study, participants were asked questions about their age, gender, sexual orientation, their general approach to relationships, the participant’s relationship with their partner, and their metamour. Participants were also asked to rate their relationship satisfaction with one of their current intimate partners (the one they chose to think about while answering questionnaires) on an 11-point scale (0–10). Higher scores indicated greater satisfaction.

### Classifying Our Metamour/Partner Emotional Response Scale (COMPERSe)

The COMPERSe (Flicker et al., 2021) is a three-factor, 11-item Likert-type self-report measure that assesses compersion conceptualized as one’s feelings about a specific partner–metamour relationship or potentially new intimate connection(s) of a given partner. The subscales measure: (1) sexual arousal to one’s partner relationship with metamour (Sexual Arousal; SA), (2) joy and contentment of one’s partner relationship with metamour (Happiness about Partner/Metamour Relationship; HPMR), and (3) excitement and thrill about the perspective of a new intimate relationship or sexual encounter of one’s partner (Excitement for New Connections, ENC). The items were rated on a 5-point scale ranging from 1 (Not at all) to 5 (A lot). Higher scores indicate higher levels of compersion.

For the current study, the Polish translation of the COMPERSe was developed. The original English version was translated into Polish by an independent translator and then back-translated by another. The back-translated version was presented to the authors of the original version. Together, several controversies related to the translation of certain phrases were agreed upon, and after a small review of item 4, the final translation received the approval of the original authors. The Polish name for the COMPERSe was chosen to be KOMPERSe, which is, like in the original version, an acronym for Kwestionariusz Oceny Metamour/Partnera: Emocje, Relacje, Seksualność. The translation of each item is presented in Table 2.

**Table 2:** Translation of items

Item	COMPERSe (English)	KOMPERSe (Polish)
1^a^	I experience sexual arousal thinking about my partner and metamour together	Doświadczam pobudzenia seksualnego, gdy myślę o tym, że mój partner i metamour są razem
2	I cherish my partner and metamour’s relationship	Cenię relację mojego partnera i metamoura
3^a^	I am delighted that my partner has a relationship with my metamour	Sprawia mi radość fakt, że mój partner jest w relacji z moim metamourem
4	I’m excited when my partner is talking to a potential hookup	Ekscytuję się, gdy mój partner z kimś flirtuje
5^a^	I feel sexual excitement when I think about my partner and metamour together	Kiedy myślę o tym, że mój partner i metamour są razem, czuję seksualną ekscytację
6^a^	I am grateful that my partner has my metamour as a partner	Czuję wdzięczność, że mój metamour jest w relacji z moim partnerem
7^a^	My partner and metamour’s relationship turns me on sexually	Relacja mojego partnera i metamoura podnieca mnie seksualnie
8	When my partner has a crush and I see that crush responding to my partner, I’m thrilled	Czuję podekscytowanie, gdy mój partner jest zauroczony kimś innym z wzajemnością
9^a^	I am very pleased that my partner and metamour are together	Bardzo się cieszę, że mój partner i metamour są razem
10	I share in the emotional high when my partner tells me about a new potential intimate partner	Cieszę się szczęściem mojego partnera, kiedy opowiada mi o swojej nowej potencjalnej intymnej relacji
11*	I am reassured knowing that my partner has my metamour as a partner	Dodaje mi otuchy myśl, że mój partner ma mojego metamoura za partnera

^a^Items that remained in the Polish version of the COMPERSe

The instruction for the tool differed between individuals who declared to be in CNM–CM relationships. It was done for obvious reasons, as CM individuals might not fully grasp the concept of consensual non-monogamy, and even less so the idea of a metamour.

Instruction for CNM participants:

Think about one of your current partners and one of their current partners, which is your metamour. If necessary, familiarize yourself with the following definition: Metamour—a person who is the partner of our partner, with whom we ourselves are not in an intimate relationship. Please fill out the following questionnaire with this situation in mind.

Instruction for monogamous participants:

In monogamous relationships, one only dates one person. However, for the purposes of this study, we will ask you to imagine a situation where your partner, with your consent, is romantically and sexually involved with someone else in addition to you. In the terminology of non-monogamous relationships, this person (your partner's partner) is called a metamour. If necessary, familiarize yourself with the following definition: Metamour—a person who is the partner of our partner, with whom we ourselves are not in an intimate relationship. Please fill out the following questionnaire with this situation in mind.

### Divergent Validity Measures

To analyze the COMPERSe validity, we used measurement tools designed to measure constructs we mentioned in the hypotheses. We assumed either positive or negative weak to medium correlations to meet criteria for both convergent and divergent validity, with the correlation coefficients about 0.3 (or  − 0.3) indicating moderate validation, and the correlation coefficients about 0.5 (or  − 0.5) indicating large validation (cf. Aithal & Aithal, 2020).

To assess jealousy, we used the questionnaire constructed by Hupka et al. (1985), named Kwestionariusz Stosunków Interpersonalnych (KSI; “Interpersonal Relations Questionnaire”) in Polish. According to Szostek (2017), it is the only standardized jealousy measurement tool used in Polish research. The Polish version of KSI (Zaleski & Hupka, 1991) is a 34-item Likert-type self-report questionnaire, in which each item was evaluated on a 7-point scale (with a missing midpoint of 4) ranging from 1 (Strongly disagree) to 7 (Strongly agree). Higher scores indicate higher jealousy. As Flicker et al. (2021) did in their research, we changed gendered language for some items (“members of the opposite sex”) to gender neutral language (“someone else”). Factor analyses on the Polish population (Zaleski & Hupka, 1991) revealed four factors of jealousy: (1) Threat to Relationship Exclusivity, (2) Self-abuse/Envy, (3) Dependence, and (4) Aversion to Partner's Autonomy. However, the study by Zaleski and Hupka (1991) did not provide any information on the reliability of subscales. Thus, we report possibly the first such analysis. Cronbach’s alphas for each of the subscales, respectively, were 0.82, 0.80, 0.69, and 0.74 for the CNM subsample and 0.86, 0.86, 0.73, and 0.78 for the CM subsample. Though Cronbach’s alpha for Dependence in the CNM subsample was slightly below the cut-point value (which is usually considered to be 0.70; cf. Tavakol & Dennick, 2011), we decided to use it in further analyses—given that Dependence is comprised of four items, and that Cronbach’s alpha indicator is usually low and underestimated for small number of items (Graham, 2006; Tavakol & Dennick, 2011), we found that near-cutoff value to be acceptable for our research. However, all the results obtained with this subscale must be treated with caution.

The State-Trait Anxiety Inventory, Form X2 (STAI-X2; Spielberger et al., 1970; Polish adaptation: Wrześniewski et al., 2002), is a 20-item self-report measure of trait anxiety. Items were rated on a 4-point Likert-like scale from 1 (Rare) to 4 (Often). A higher score indicates a higher level of trait anxiety. Cronbach’s alphas were 0.85 for the CNM subsample and 0.87 for the CM subsample.

The Kwestionariusz Stylów Przywiązaniowych (KSP; “Attachement Style Questionnaire”; Plopa, 2008) is a three-factor, 24-item, Likert-type self-report scale that allows the assessment of different attachment styles: (1) Secure, (2) Anxious-ambivalent, and (3) Avoidant. Individuals responded to each of the statements on a 7-point scale ranging from 1 (Strongly disagree) to 7 (Strongly agree). The higher the score on each subscale, the higher the tendency to be securely, anxiously, or avoidantly attached. As we noted before, we assumed subscales 2 and 3 to be indicators of divergent validity and subscale 1 of convergent validity. Cronbach’s alphas for the CNM and CM subsamples were 0.81 and 0.85 for Secure style, 0.85 and 0.89 for Anxious-ambivalent style, and 0.83 and 0.80 for Avoidant style, respectively.

### Convergent Validity Measures

The Interpersonal Reactivity Index for Couples (IRIC; Péloquin & Lafontaine, 2010) in Polish adaptation (Kaźmierczak & Karasiewicz, 2021) is a two-factor, 10-item Likert-type self-report scale assessing empathy within a dyadic intimate relationship. The subscale (1) Empathetic Concern refers to emotional empathy and (2) Perspective Taking refers to cognitive empathy. Items were rated on a 5-point Likert-like response scale from 1 (I do not agree at all—does not describe me well) to 5 (I completely agree—describes me very well). Higher scores indicate higher empathy. The questionnaire was also used in the original research (Flicker et al., 2021). Cronbach’s alphas for the CNM and CM subsamples were 0.65 and 0.62 for Empathetic Concern and 0.81 and 0.88 for Perspective Taking, respectively. Unfortunately, the reliability indexes for Empathetic Concern were below even the low cut-point of 0.70 (cf. Tavakol & Dennick, 2011). Thus, we decided not to include this subscale in any of the further analyses.

The Positive Mood scale of the Positive and Negative Affect Scale (PANAS; Watson et al., 1988; Polish adaptation: Brzozowski, 2010) is a 10-item Likert-type scale assessing the tendency to experience positive affect. Individuals rated how they felt in general on a scale that ranged from 1 (very slightly or not at all) to 5 (extremely). The higher the score, the greater the tendency to experience positive emotions. The questionnaire was also used in the original research (Flicker et al., 2021). Cronbach’s alphas were 0.78 and 0.74 for the CNM and CM subsamples, respectively.

The Emotional Intelligence Questionnaire (Schutte et al., 1998; Polish adaptation: Jaworowska & Matczak, 2008; INTE) is a 33-item Likert-type self-report scale that assesses emotional intelligence (EI), with a range of responses from 1 (I don’t agree at all) to 5 (I completely agree). A higher score indicates a higher EI. Cronbach’s alphas were 0.88 and 0.86 for the CNM and CM subsamples, respectively.

The Self-liking/Self-competence Scale-Revised (SLCS-R; Tafarodi & Swann, 2001; Polish adaptation: Szpitalak & Polczyk, 2015) is a two-factor, 16-item Likert-type self-report measure of two factors that assesses one’s self-esteem based on two correlated dimensions: (1) Self-liking, which measures feelings of social worth, and (2) Self-competence, which measures feelings of efficacy and control. The overall score can also be calculated by adding the scores of both subscales. Items were rated on a 5-point Likert-like response scale from 1 (strongly disagree) to 5 (strongly agree). A higher score indicates a higher self-esteem. Cronbach’s alphas for the CNM and CM subsamples were 0.89 and 0.91 for Self-liking, 0.78 and 0.78 for Self-competence, and 0.90 and 0.90 for the overall score, respectively.

### Procedure

The study was conducted using Qualtrics (Provo, UT). Participants first read the description of the research information approved by the Research Ethics Committee, provided consent, and agreed to participate. After completing demographic and relationship information, CNM individuals were instructed to choose one current intimate partner who had at least one current relationship with another partner and to think about them for all questions throughout the study. On the other hand, CM individuals were instructed to think about their current partner when answering questions. The questionnaires were presented in randomized order. The entire study took approximately 20 min to complete. After answering all questions, participants were asked to provide an email address to resend the COMPERSe questions four weeks later to assess test–retest reliability.

## Results

### Factor Structure

Confirmatory factor analysis (CFA) using a maximum likelihood estimator was performed using lavaan software (Rosseel, 2012) running under R Environment (R Core Team, 2016). The CFA sample size consisted of all 211 participants from the CNM subsample. Although this sample did not reach a similar value to the original study, it still exceeded the recommendations from Worthington and Whittaker (2006) of 5 to 10 participants per parameter to be estimated and was also sufficient based on the sample size calculations mentioned in the Participants section.

The results of CFA on the full-length 11-item three-factor scale are presented in the first row of Table 3. Based on interpretations by Hu and Bentler (1999), we assumed following values of fit indices as acceptable: Comparative Fit Index (CFI): > 0.95, Tucker–Lewis Index (TLI): > 0.95, root mean square error of approximation (RMSEA): < 0.06 (or < 0.08), and standardized root mean square residual (SRMR): < 0.08. The fit indices TLI and RMSEA for the preliminary Polish version turned out to be not satisfactory, being 0.94 and 0.10, respectively. To improve the fit of the model, we verified the standardized factor loadings, but all were above 0.7, exceeding the recommendations by Tabachnick and Fidell’s (2007) and Comrey and Lee’s (1992) interpretation of more stringent cutoffs (in which 0.63 indicates very good value). Examining modification indices for factor loadings, we found that allowing questions 2, 4, and 10 to load also the Sexual Arousal (SA) factor, and item 10 to load also Happiness about Partner/Metamour Relationship (HPMR) factor would significantly improve the three-factor model’s fit, *Mi*s > 12.96. Related to this, for most items cross-loadings’ values were very low, i.e., between  − 0.05 and 0.07. However, items from ENC scale cross-loaded also SA scale (item 4: 0.35; item 8:  − 0.10; item 10:  − 0.28) and HPMR scale (item 4:  − 0.31; item 8:  − 0.19; item 10: 0.52). Additionally, item 2 from HPMR scale also cross-loaded SA scale ( − 0.17). Furthermore, ENC scale demonstrated very high positive correlation with HPMR (0.76) and high with SA (0.54) (for comparison, correlation between HPMR and SA was 0.37). As we did not find it sensible to make some items load several subscales, and due to high covariances of ENC with other scales, we therefore decided to perform an exploratory factor analysis to identify whether the factor structure of the Polish version of the COMPERSe differs from the original. Using the split file function in SPSS (IBM, 2021), we randomly split the CNM sample into two in proportion 1:2, creating an exploratory factor analysis subsample (EFA; *n* = 69) and a confirmatory factor analysis subsample (CFA; *n* = 142). Due to the proportion used, the size of the CFA subsample was still sufficient according to the recommendations mentioned previously. We performed principal axis factoring (PAF) with varimax rotation using SPSS version 28.0 (IBM, 2021) in the EFA subsample. The KMO Measure of Sampling Adequacy = 0.87 and Bartlett’s Test of Sphericity *χ*^*2*^(df = 55) = 741.14, *p* < 0.001, indicated that the subsample was suitable for the EFA (cf. Tabachnick & Fidell, 2001; Worthington & Whittaker, 2006).

**Table 3:** Fit indices for all confirmatory factor analyses

Model	ML χ^2^	df	*p*	CFI	TLI	SRMR	RMSEA	CIs for RMSEA	
								L 90% CI	H 90% CI
Three-factor (original)	126.94	41	< .001	0.96	0.94	0.06	0.10	0.08	0.12
Two-factor (revised)	19.07	13	.121	0.99	0.99	0.03	0.06	< 0.01	0.11
One-factor	364.01	14	< .001	0.60	0.40	0.26	0.42	0.38	0.46

The results of EFA are presented in Table 4. Only two factors had eigenvalues greater than 1; therefore, we assumed that Polish version of the COMPERSe differs from the original version. Five items (2, 4, 6, 8, 10) were considered for removal due to communality below the recommended 0.7 cutoff ideal value (Beavers et al., 2013). This included all items from Excitement for New Connections scale (ENC; 4, 8, 10), which at the same time had low loadings and simultaneously high cross-loadings with two remaining factors (for items 4, 8, and 10: 0.54 vs. 0.58, 0.67 vs. 0.36, 0.71 vs. 0.26, for Factor 1 vs. Factor 2, respectively). As can be seen, most of the problematic items were those that would improve the fit of the three-factor model if they load more than one subscale. Considering these results, as well as previous CFA, we decided to eliminate all items from the ENC subscale (thus removing the entire subscale) and Item 2 but leaving Item 6 due to its relative high communality (slightly below the 0.7 cutoff) and—contrary to Item 2—due to lack of suggestion that this item would load more than one subscale. Therefore, it can be considered that in the Polish version of the COMPERSe the two-factor 7-item structure ((1) Sexual Arousal, SA; (2) Happiness about Partner/Metamour Relationship, HPMR) is the most cohesive, explaining 77.24% of the variance in the COMPERSe scores.

**Table 4:** Eigenvalues, % variance explained, and cumulative % variance explained

Factor	Eigenvalue	% of variance explained by factor	Cumulative % of variance explained
1	5.97	54.27	54.27
2	2.53	22.98	77.24
3	.92	8.33	85.57
4	.37	3.39	88.95
5	.33	3.03	91.98

Eigenvalues ≥ .30 listed

**Fig. 1:**
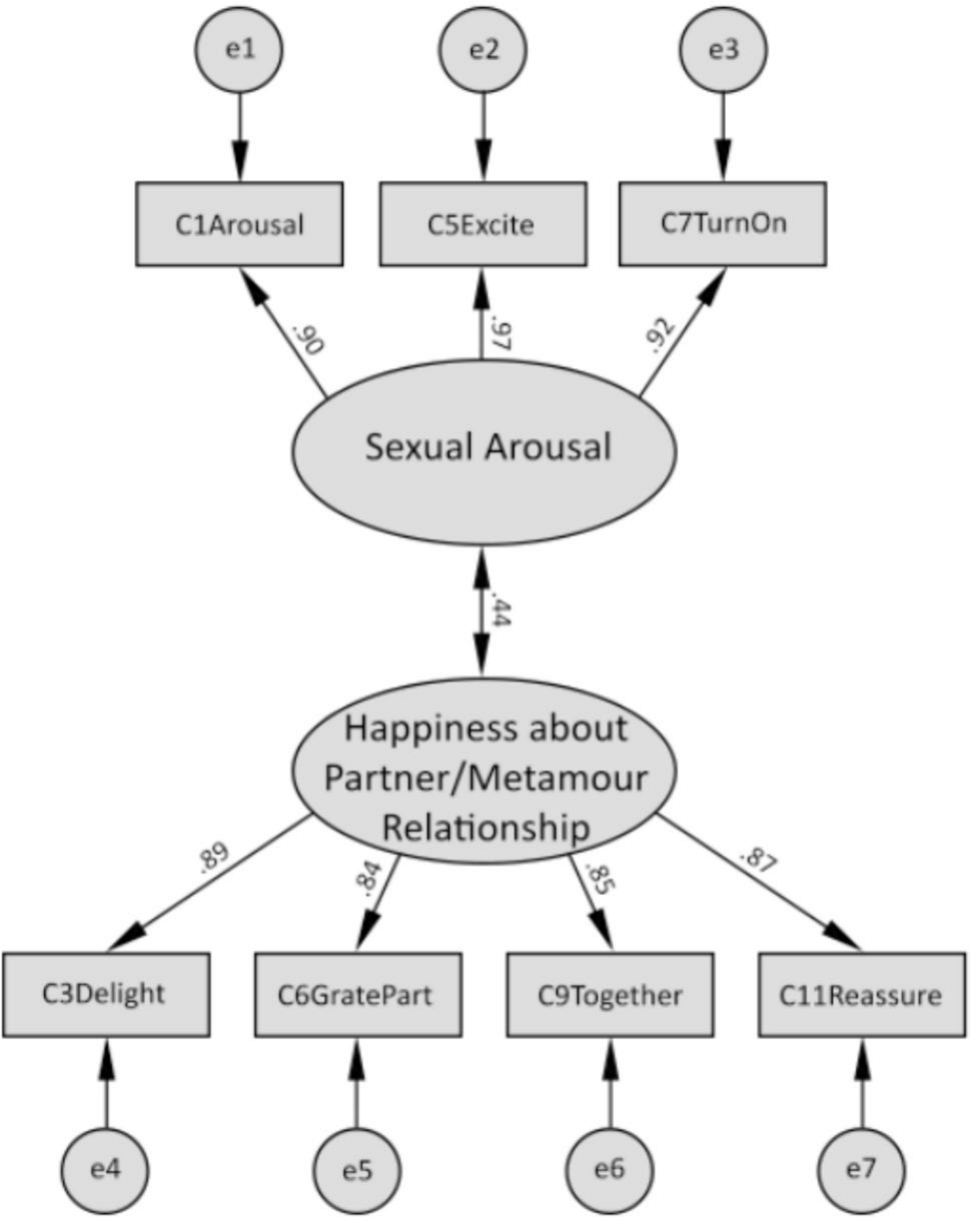
Results of the confirmatory factor analysis. *Note* Figure displays the results of the structural equation model including factor loadings of items and correlations between the factors. The names of the items are taken from the original research (Flicker et al., 2021). *χ*2 (13, *N* = 142) = 19.07, *p* = 0.121, Goodness of fit (GFI) = 0.96, Normed fit index (NFI) = 0.98, Incremental fit index (IFI) = 0.99, Tucker–Lewis index (TLI) = 0.99, Comparative fit index (CFI) = 0.99; SRMR = 0.06, RMSEA = 0.06 [90% CI (<0.01–0.11)]. e = error

Subsequently, we performed a new CFA to determine whether the data fit the two-factor model emerging from the EFA. As mentioned above, the size of the CFA subsample was 142. The two-factor model provided an excellent fit (cf. Hu & Bentler, 1999). The chi-square value was statistically insignificant (13, *N* = 142) = 19.07, *p* = 0.121, and the CFI, TLI, RMSEA, and SRMR were 0.99, 0.99, 0.06, and 0.03, respectively. The two-factor model was then compared with the one-factor model. The one-factor model did not meet any of the goodness-of-fit criteria: The chi-square value was statistically significant (14, *N* = 142) = 364.01, *p* < 0. 1, and CFI, TLI, RMSEA, and SRMR were 0.60, 0.40, 0.42, and 0.26, respectively. The results of the previous and new CFAs are shown in Table 4 (row 1 and 2, respectively), and the results of the new CFA of the two-factor model are also shown in Fig. 1. All standardized factor loadings for the two-factor model of the COMPERSe exceeded the value of 0.80 and are presented in Table 5. For further analyses, we have used this shortened 7-item, two-factor Polish version of the COMPERSe instead of original full-length US version to better reflect the structure of conceptualizing compersion in the Polish CNM community. The reasons of the differences between structures of US and Polish versions of the tool will be discussed in the Discussion section relevant paragraph.

**Table 5:** Scale items with factor loadings

Item	EFA subsample		CFA subsample	
	1	2	1	2
*Factor 1: Happiness about partner/metamour relationship*				
3. Sprawia mi radość fakt, że mój partner jest w relacji z moim metamourem	.93		.89	
6. Czuję wdzięczność, że mój metamour jest w relacji z moim partnerem	.80		.84	
9. Bardzo się cieszę, że mój partner i metamour są razem	.94		.85	
11. Dodaje mi otuchy myśl, że mój partner ma mojego metamoura za partnera	.85		.87	
*Factor 2: Sexual arousal*				
1. Doświadczam pobudzenia seksualnego, gdy myślę o tym, że mój partner i metamour są razem		.92		.90
5. Kiedy myślę o tym, że mój partner i metamour są razem, czuję seksualną ekscytację		.95		.97
7. Relacja mojego partnera i metamoura podnieca mnie seksualnie		.94		.92

### Reliability and Test–Retest Stability

Cronbach’s alphas for both subscales in the Polish version of the COMPERSe were very high, above 0.9 for both CNM and CM samples (see Table 6), indicating excellent internal consistency.

**Table 6:** Means, SDs, Cronbach’s alphas, and correlations between subscales and validity measures

Variables	CNM sample		CM sample	
	CS-HPMR	CS-SA	CS-HPMR	CS-SA
Means and (SDs)	3.60 (1.04)	2.50 (1.24)	2.06 (1.19)	1.82 (1.06)
Cronbach’s alphas	0.93	0.95	0.96	0.95
*Correlations between subscales*				
CS-SA	0.37***	–	0.63***	–
*Divergent validity*				
Jealousy				
Threat to relationship exclusivity	−.50***	−.05	−.59***	−.46***
Self-abuse/envy	−.28***	−.10	.12	.20*
Dependence	−.09	.16*	−.13	.02
Aversion to partner’s autonomy	−.51***	−.22**	−.40***	−.23**
Anxiety (trait)	−.23**	−.12	.04	−.07
Anxious-ambivalent Attachment Style	−.36***	.04	−.12	−.17
Avoidant Attachment Style	−.20**	−.05	.06	.05
*Convergent validity*				
Secure Attachment Style	.32***	.13*	−.17	−.23*
Perspective Taking	.32***	.19**	−.11	−.12
Positive Mood	.10	.10	.08	.09
Emotional Intelligence	.18*	.12	−.12	−.16
Self-esteem	.17*	.05	−.08	.07
Self-liking	.16*	.04	0.05	.06
Self-competence	.14*	.05	−.10	.05
Relationship satisfaction	.32***	.14*	.02	.01

*CS-HPMR* Compersion scale, happiness about partner/metamour relationship; *CS-SA* Compersion scale, sexual arousal subscale

*p < .05, **p < .01, ***p < .001

To obtain test–retest stability, we calculated the intra-class correlation coefficients (ICC) using SPSS software version 28.0 (IBM, 2021). The minimum sample size for ICC was determined using the Sample Size Calculator (web) (Arifin, 2022; cf. Arifin, 2018). The minimum sample size required for ICC was indicated to be 80, with the minimum acceptable reliability set at 0.60, a cutoff value (Cicchetti, 1994), expected reliability = 0.75, considered the lowest value indicative of good reliability (Koo & Li, 2016), 2 repetitions, one-tailed test with power = 0.80. Eventually, 92 participants assessed the test after four weeks delay (83 from CNM samples and 9 from CM samples). The ICC used a mixed two-way model, in absolute agreement with 95% CI. The values were excellent: 0.94, 95% CI [0.91–0.96] for both HPMR and SA.

### Divergent and Convergent Validity

The divergent and convergent validity was examined separately in the CNM and CM samples. The correlations between the variables are presented in Table 6.

#### Consensually Non-Monogamous Sample

In general, the hypotheses were confirmed. In the case of divergent validity, jealousy was negatively related to the HPMR scale: moderately with Threat to Relationship Exclusivity and Aversion to Partner’s Autonomy, and weakly with Self-abuse/Envy, but was not correlated with Dependence. Meanwhile, the SA scale was negatively and weakly correlated with Aversion to Partner’s Autonomy and, surprisingly, weakly and positively with Dependence. Furthermore, the HPMR scale was negatively and rather weakly related to trait anxiety and two attachment styles: anxious and avoidant.

In the case of convergent validity, the HPMR scale was positively and weakly related to secure attachment style, cognitive empathy (Perspective Taking), emotional intelligence, self-esteem (overall and both subscales), and relationship satisfaction. The SA scale, in turn, did not correlate with most measures, although we observed positive but weak correlations with secure attachment style, cognitive empathy, and relationship satisfaction. Therefore, most of the results match those of the original study (Flicker et al., 2021) when the same or similar tools were used.

In summary, the general evidence for the validity of the COMPERSe scores was fairly strong. As in the study by Flicker et al. (2021), the HPMR scale had the most consistent relationships with the validity variables, while the correlations between the SA scale and the validity variables were somewhat weaker.

#### Consensually Monogamous Sample

Regarding the CM sample, we did not make any predictions beforehand; thus, the results reflect mostly exploratory analyses. We observed fewer significant relationships between compersion and measured variables. Like the CNM sample, Threat to Relationship Exclusivity and Aversion to Partner’s Autonomy were negatively and weakly to moderately related to both of the COMPERSe scales. The major difference was the moderate negative correlation between SA and Threat to Relationship Exclusivity, which was not observed in CNM sample. Moreover, we observed a weak but positive correlation between the SA scale and Self-abuse/Envy. Other divergent validity measures did not correlate with any of the COMPERSe scales in the CM sample.

The relationship between compersion and convergent validity measures was almost nonexistent. Only one weak and negative correlation was observed: SA with secure attachment style.

#### Consensually Non-Monogamous and Consensually Monogamous Differences

In addition to the adaptation of the COMPERSe, we wanted to test other hypotheses, especially about the differences between the CNM and monogamous participants. We expected a higher level of compersion (both HPMR and SA scales) and lower levels of jealousy among CNM individuals compared to monogamous individuals. Furthermore, we also examined whether these two groups differ in other measured variables.

An independent samples *t*-test was performed using SPSS version 28.0 (IBM, 2021). We confirmed our hypothesis that CNM individuals would experience higher compersion than their monogamous peers, considering both HPMR (*t*(337.02) = 13.27, *p* < 0.001, *d* = 1.38) and SA (*t*(376.39) = 5.68, *p* < 0.001, *d* = 0.58). Likewise, monogamous individuals were characterized by a higher level of jealousy on almost all scales (excerpt Dependence, where the difference was consistent with the direction of the hypothesis, but on the verge of statistical significance)—*t*s(≤ 250.16) ≤  − 9.35, *p*s ≤ 0.002, *d*s ≤ 1.10. It was also revealed that CNM individuals exhibited anxious attachment style traits less often than monogamous people (*t*(214.40) =  − 2.84, *p* = 0.005, *d* = 0.35) and that CNM people are characterized by higher levels of cognitive empathy than their monogamous peers (*t*(274) = 2.68, *p* = 0.008, *d* = 0.32). Finally, it turned out that CNM individuals were generally more satisfied with their relationships than monogamous individuals (*t*(278.58) = 3.77, *p* < 0.001, *d* = 0.40). All analyses are summarized in Table 7.

**Table 7:** Mean scores on measures for consensually non-monogamous (CNM) and consensually monogamous (CM) participants and differences

Variables	CNM sample		CM sample		*t*(df^a^)	*p*	Cohen’s *d*
	M	SD	M	SD			
*Compersion*							
CS-HPMR	3.60	1.04	2.06	1.19	13.27^1^	< 0.001	1.38
CS-SA	2.50	1.24	1.82	1.06	5.68^2^	< 0.001	0.58
*Jealousy*							
TRE	2.36	0.79	3.34	1.00	−9.35^3^	< 0.001	1.10
SAE	2.27	0.84	2.61	0.98	−3.16^4^	0.002	0.37
D	2.71	1.04	2.93	1.06	−1.83^5^	0.068	0.21
APA	1.62	0.54	2.12	0.72	−6.74^6^	< 0.001	0.79
Anxiety, trait	2.48	0.49	2.51	0.51	−0.43^7^	0.669	0.06
*Attachment styles*							
Secure	5.74	0.94	5.57	1.11	1.38^8^	0.168	0.17
Anxious-ambivalent	3.02	1.22	3.48	1.44	−2.84^9^	0.005	0.35
Avoidant	2.15	1.01	2.30	1.04	−1.19^10^	0.236	0.14
*Empathy*							
Perspective Taking	4.06	0.57	3.85	0.68	2.68^11^	0.008	0.32
Positive Mood	3.39	0.55	3.21	0.48	1.61^12^	0.109	0.20
Emotional intelligence	3.64	0.45	3.63	0.40	0.26^13^	0.795	0.03
Self-esteem	2.87	0.69	2.77	0.65	1.16^14^	0.249	0.15
Self-liking	2.99	0.90	2.81	0.90	1.55^15^	0.123	0.20
Self-competence	2.76	0.62	2.74	0.57	0.29^16^	0.773	0.04
Relationship satisfaction	8.16	1.76	7.27	2.66	3.77^17^	< 0.001	0.40

*CS-SA* Compersion scale, sexual arousal subscale; *CS-HPMR* Compersion scale, happiness about partner/metamour relationship; *TRE* Threat to relationship exclusivity; *SAE* Self-abuse/envy; *D* Dependence; *APA* Aversion to Partner’s Autonomy

^a^dfs for each comparison: ^1^337.02; ^2^376.39; ^3^234.51; ^4^250.16; ^5^309; ^6^227.10; ^7^255; ^8^214.10; ^9^214.40; ^10^236.82; ^11^274;^12^272; ^13^261; ^14^255; ^15^255; ^16^255; ^17^278.58

#### Open Relationships and Polyamory Differences

Besides the differences between CNM and monogamous people, we also expected some differences between people involved in different types of CNM relationships. To assign participants to groups, we used the declaration about the type of relationship they have with one of their current intimate partners (the one they chose to think about while answering questionnaires). Unfortunately, the only two types of relationships that had a sufficient sample size to perform analyses were polyamory and open relationships (*n*s ≤ 70). Therefore, we only examined the differences between the two groups. We expected a higher level of HPMR, but not SA among polyamorous individuals compared to people in open relationships. Furthermore, we also examined whether these two groups differ in other measured variables.

An independent samples *t*-test was performed using SPSS version 28.0 (IBM, 2021). The hypothesis that polyamorous individuals would have higher levels of HPMR was confirmed (*t*(167) =  − 3.49, *p* = 0.001, *d* = 0.54). At the same time, there was no difference in SA between the two groups (*t*(167) =  − 0.91, *p* = 0.364, *d* = 0.14). It was also revealed that individuals in open relationships had a greater aversion to partner’s autonomy than polyamorous individuals (*t*(115.94) = 2.46, *p* = 0.016, *d* = 0.41). Furthermore, polyamorous individuals exhibited secure attachment style traits more often than people in open relationships (*t*(141) =  − 2.02, *p* = 0.046, *d* = 0.35). The difference concerning higher levels of cognitive empathy among polyamorous individuals was on the verge of statistical significance (*t*(136) =  − 1.88, *p* = 0.062, *d* = 0.32). Regardless of the differences, both groups declared similar relationship satisfaction. All analyses are summarized in Table 8.

**Table 8:** Mean scores on measures for consensually non-monogamous and monogamous participants and differences

Variables	Open relationship		Polyamory		*t*(df^a^)	*p*	Cohen’s *d*
	M	SD	M	SD			
*Compersion*							
CS-HPMR	3.34	1.07	3.89	0.95	−3.49^1^	0.001	0.54
CS-SA	3.36	1.23	2.54	1.27	−0.91^2^	0.364	0.14
*Jealousy*							
TRE	2.35	0.83	2.21	0.65	1.17^3^	0.243	0.19
SAE	2.31	0.89	2.12	0.73	1.43^4^	0.156	0.23
D	2.85	1.10	2.67	0.97	1.02^5^	0.308	0.17
APA	1.68	0.55	1.48	0.41	2.46^6^	0.016	0.41
Anxiety, trait	2.48	0.45	2.42	0.53	0.67^7^	0.503	0.12
*Attachment styles*							
Secure	5.68	1.01	5.99	1.06	−2.02^8^	0.046	0.35
Anxious-ambivalent	2.98	1.32	2.87	0.82	0.58^9^	0.564	0.12
Avoidant	2.11	0.90	1.95	0.72	1.08^10^	0.282	0.14
*Empathy*							
Perspective taking	3.98	0.61	4.16	0.54	−1.88^11^	0.062	0.32
Positive mood	3.40	0.60	3.42	0.52	−0.14^12^	0.889	0.02
Emotional Intelligence	3.61	0.45	3.70	0.44	−1.26^13^	0.210	0.22
Self-esteem	2.82	0.71	2.96	0.72	−1.10^14^	0.275	0.19
Self-liking	2.93	0.93	3.09	0.93	−0.98^15^	0.329	0.17
Self-competence	2.71	0.63	2.83	0.64	−1.02^16^	0.210	0.18
Relationship satisfaction	8.23	1.70	8.34	1.55	−0.46^17^	0.648	0.07

*CS-SA* Compersion scale, sexual arousal subscale; *CS-HPMR* Compersion Scale, Happiness about Partner/Metamour Relationship; *TRE* Threat to relationship exclusivity; *SAE* Self-abuse/envy; *D* Dependence; *APA* Aversion to Partner’s Autonomy

^a^dfs for each comparison: ^1^167; ^2^167; ^3^147; ^4^147; ^5^147; ^6^115.94; ^7^127; ^8^141; ^9^141; ^10^107.81; ^11^136; ^12^121; ^13^130; ^14^127; ^15^127; ^16^127; ^17^267

#### Mediation Analysis

Another issue worth considering is the role of compersion in relationship satisfaction among CNM people. Since all variables (except Dependence subscale of Jealousy) showed significant correlations with relationship satisfaction from small to high strength, we ran a single regression analysis using all these variables. It turned out that only three variables predicted relationship satisfaction independently from others: Aversion to Partner’s Autonomy (*β* =  − 0.24, *p* = 0.007), secure attachment style (*β* = 0.30, *p* = 0.015) and positive mood (*β* = 0.19, *p* = 0.027). This meant that the relationship between compersion and relationship satisfaction was not direct and was probably mediated by some other variable. From a theoretical perspective, a potentially likely mediator could be jealousy. This claim finds support in the literature—Flicker et al. (2021) speculated that compersion may mitigate the experience of jealousy and temper it, consequently increasing relationship satisfaction. Therefore, two mediation analyses were performed using PROCESS v. 4.1 software (Model 4; Hayes, 2022), where, in both cases, Aversion to Partner’s Autonomy was a mediator and where (1) HPMR was a predictor and SA was a covariate, and (2) SA was a predictor and HPMR was a covariate.

**Fig. 2:**
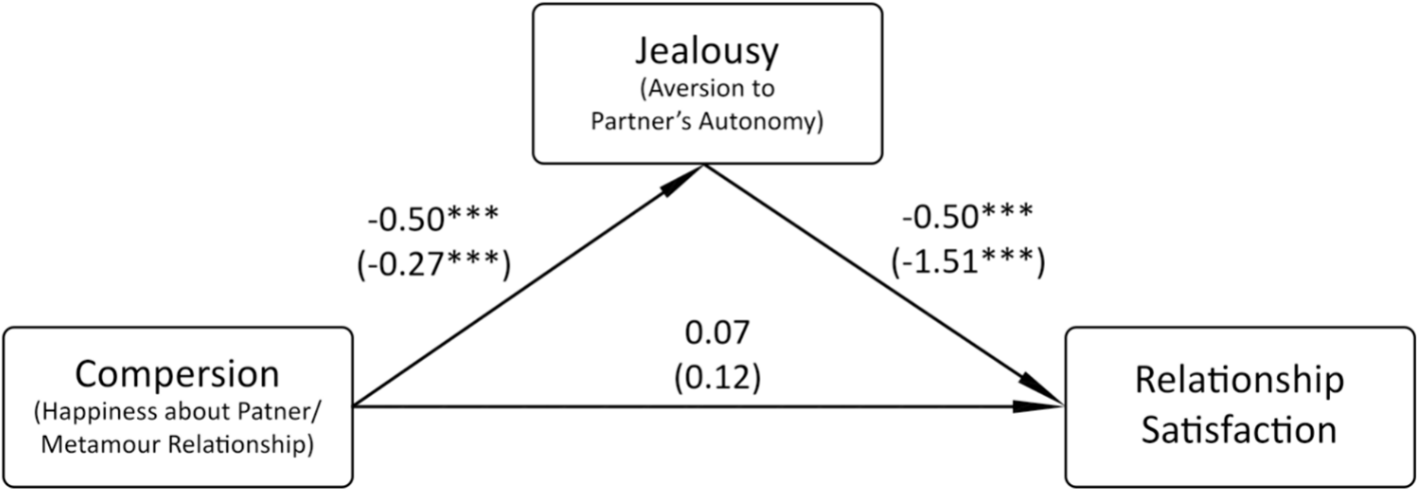
Results of the first mediation analysis; model of compersion, jealousy and relationship satisfaction. Standardized coefficients are presented first, followed by unstandardized coefficients in parentheses. *p < 0.05, **p < 0.01, ***p < 0.001

Since not all participants responded to all questionnaires, the final sample for both analyses consisted of 182 individuals. The indirect effect on Relationship Satisfaction turned out to be significant only for HPMR (B = 0.41 SE = 0.13, 95% CI [0.17, 0.66]), which suggests that HPMR increases relationship satisfaction by decreasing aversion to partner’s autonomy. The model is presented in Fig. 2.

#### The Role of Cognitive Empathy

Furthermore, we were interested in examining the role of possible facilitators of compersion in the mediation model described in the previous paragraph. It is an especially important issue because, as detailed by Flicker et al. (2021), understanding the factors that facilitate compersion has important implications for relationship therapy for non-monogamous people. Firstly, compersion is often identified as an ideal among polyamorous people, but many struggle with experiencing it (Deri, 2015; Ferrer, 2008); therefore, knowing of factors that can stimulate feelings of compersion could help people who are motivated to experience it. Secondly, understanding the factors that facilitate compersion could contribute to the development of new strategies to manage jealousy, which can negatively contribute to relationship satisfaction.

The factor we found promising was cognitive empathy. A recent study (Flicker et al., 2022) suggested that cognitive empathy could be a factor that facilitates compersion. Furthermore, empathy may be learned during training. Teding van Berkhout & Malouff, 2016 in their meta-analysis noticed that all studies they analyzed focused on at least cognitive empathy, because it is considered to involve processes that can be consciously acquired, contrary to emotional or behavioral empathy (cf. Batt-Rawden et al., 2013). Therefore, if cognitive empathy can predict compersion, training techniques that increase cognitive empathy would be a valuable contribution to potential new compersion training techniques that can increase satisfaction in CNM relationships.

**Fig. 3:**
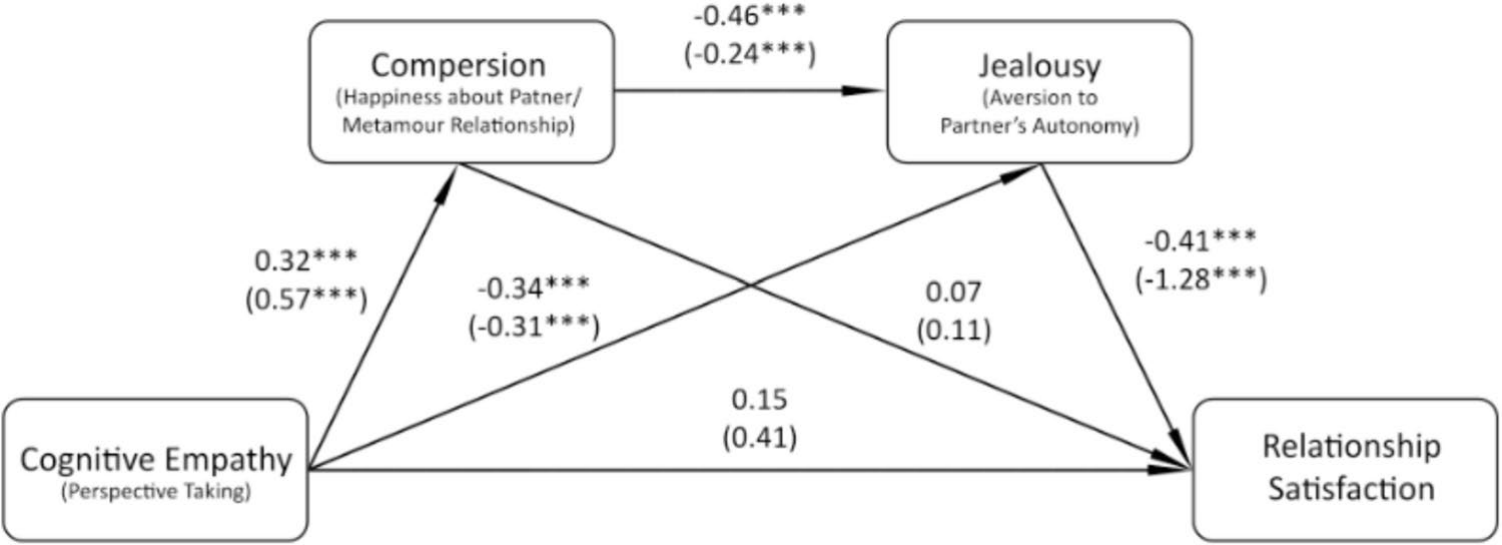
Results of the second mediation analysis; general model of cognitive empathy, compersion, jealousy and relationship satisfaction (ECJS). Standardized coefficients are presented first, followed by unstandardized coefficients in parentheses. *p < 0.05, **p < 0.01, ***p < 0.001

We examined whether cognitive empathy predicted compersion and whether it translated to higher relationship satisfaction both directly and through mediators. We tested serial mediation Model 6, which is presented in Fig. 3. Since not all participants responded to all questionnaires, the final sample consisted of 165 individuals. The overall total effect was significant (B = 1.03, SE = 0.21, 95% CI [0.63, 1.44]). According to the predictions, the main indirect effect of cognitive empathy through HPMR and jealousy was also significant (B = 0.17, SE = 0.07, 95% CI [0.04, 0.32]). Furthermore, the indirect effect of cognitive empathy through jealousy was also significant (B = 0.39, SE = 0.20, 95% CI [0.08, 0.84], while the direct effect of cognitive empathy on relationship satisfaction was not significant (B = 0.41, SE = 0.22, 95% CI [ − 0.1, 0.84]). It means that cognitive empathy can influence relationship satisfaction in two ways: (1) by facilitating compersion, which lowers the jealousy, resulting in higher satisfaction; and (2) directly decreasing jealousy.

#### Mediation Model Among Open Relationships and Polyamory

**Fig. 4:**
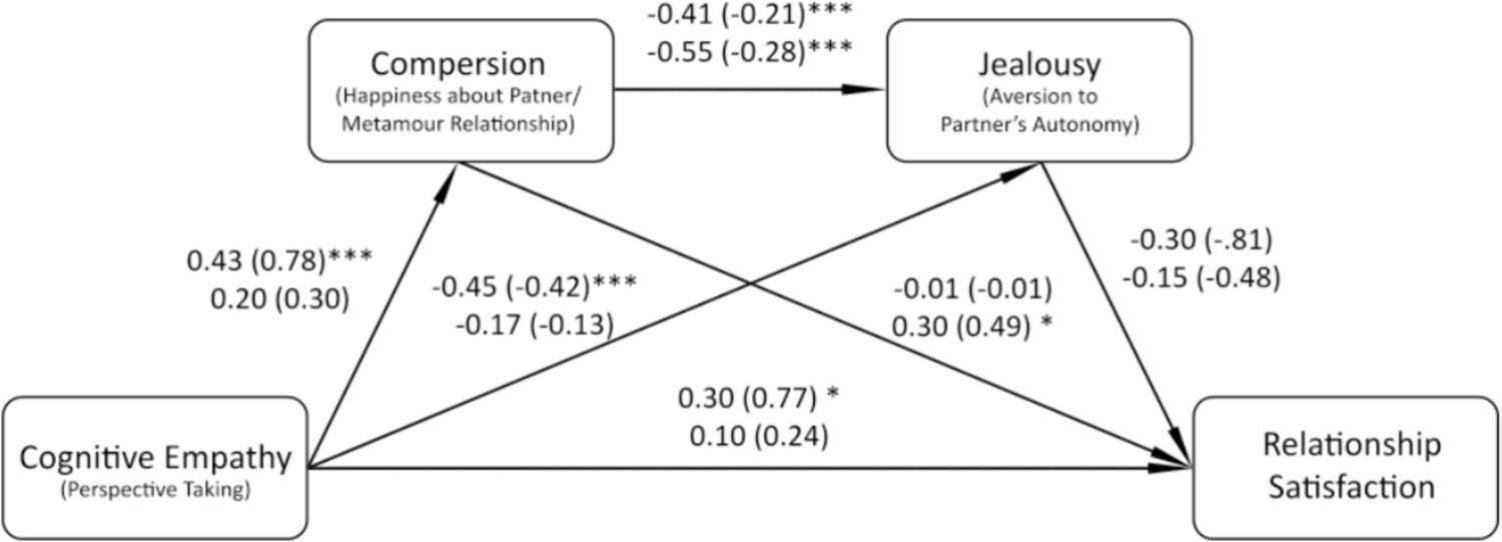
Results of the general ECJS model analysis for open relationships and polyamory. Standardized coefficients are presented first, followed by unstandardized coefficients in parentheses. For each path, first coefficients are presented for Open relationships, and second for Polyamory. *p < 0.05, **p < 0.01, ***p < 0.001

Having constructed the general model for CNM relationships, we also decided to examine whether it would fit polyamorous and open relationships differently, considering differences that were observed in compersion and jealousy, and also, to some extent, in cognitive empathy. The model is presented in Fig. 4.

For open relationships (60 individuals), total effect was significant (B = 1.24, SE = 0.30, 95% CI [0.65, 1.83]), but the indirect effect of cognitive empathy through HPMR and jealousy was not significant (B = 0.13, SE = 0.12, 95% CI [ − 0.03, 0.42]), as well as the indirect effect of cognitive empathy through jealousy (B = 0.34, SE = 0.24, 95% CI [ − 0.12, 0.84]). However, the direct path from cognitive empathy to relationship satisfaction was significant (B = 0.77, SE = 0.37, 95% CI [0.02, 1.51]). At the same time, for polyamory (78 individuals) the model turned out to be not significant (total effect: B = 0.49, SE = 0.28, 95% CI [ − 0.06, 1.04]; direct effect of cognitive empathy: B = 0.24, SE = 0.27, 95% CI [ − 0.30, 0.77]; main indirect effect: B = 0.04, SE = 0.05, 95% CI [ − 0.03, 0.18]; indirect effect of empathy through jealousy: B = 0.06, SE = 0.08, 95% CI [ − 0.06, 0.28]). However, the direct effect of HPMR was significant (B = 0.49, SE = 0.21, 95% CI [0.07, 0.91]). In both cases, the path from jealousy to relationship satisfaction was not significant, contrary to the general model that covers all types of CNM relationships. In addition, only in the open relationships group the path from cognitive empathy to HPMR was significant, but it was not significant in the polyamory group. It means that the relationships between compersion, jealousy, cognitive empathy, and satisfaction are far more complicated than we first expected and may be different when considering different types of CNM relationships separately and different when CNM relationships are collectively analyzed.

## Discussion

In this paper, we described the adaptation of the first validated quantitative measure of compersion (the COMPERSe questionnaire; Flicker et al., 2021) to the Polish-speaking population. The analyses did not support a three-factor model described in the original research; instead, revisions of the scale based on the removal of cross-loading items resulted in a 7-item, two-factor model representing two out of three themes from the original model: (1) Happiness about the Partner/Metamour Relationship (HPMR) and (2) Sexual Arousal to the partner/metamour relationship (SA). Both subscales demonstrated excellent internal consistency, strong divergent and convergent validity, and excellent test–retest stability.

### Structure of the Polish Version of COMPERSe

Our research suggested that in the Polish version of COMPERSe ENC items were not only not able to establish independent subscale, but also had relatively high cross-loadings with two remaining factors. One reason why all the items from Excitement for New Connections (ENC) subscale did not fit to the entire questionnaire's three-factor or even two-factor structures, may be that the translation of the items did not fully reflect their original meaning (even though the back-translation did meet with the approval of the authors of the COMPERSe). However, it does not explain well enough why all ENC items would have been mistranslated, and others were not.

The differences may also be due to some cultural differences between Polish people and predominantly Americans, who were the subjects in the study by Flicker et al. (2021). Although Poland is considered a Western country, the differences between it and other Western countries regarding relationship coping and satisfaction can vary (Hilpert et al., 2016). This may represent different mindsets, also affecting the perception of consensual non-monogamy and, in consequence, the excitement for potentially new metamour or partner’s potential hookup. It is possible that for Polish CNM people, the feelings of emotional excitement, thrill or joy were not too distinctive from experiencing either sexual arousal or emotional happiness, or were perceived as a mix of both, regardless of the length or status of the partner–metamour relationship. However, these speculations require further research.

Another explanation is that this study was conducted shortly after COVID-19 restrictions were being lifted in Poland in 2022, allowing CNM individuals the opportunity to date new people again. While the data were collected when quarantine measures were not in place, it is possible that the prolonged effects of COVID-19 restrictions could have influenced the perception of excitement for connections with new metamours among CNM individuals, thus affecting their responses. This could have impacted the variability and factor loadings, subsequently causing issues with the ENC subscale. To investigate this possibility, we suggest that a new validation study may be necessary in the coming years when the impact of quarantine restrictions may not be as significant.

Flicker et al. (2021) speculated that the ENC and HPMR scales reflect the difference between New Relationship Energy (NRE; excitement and joyfulness) and Established Relationship Energy (ERE; comfort, security and stability) (cf. Stewart, 2001), respectively. When one’s partner has NRE or ERE toward their partner, these feelings can be mirrored as NRE or ERE toward one’s partner’s and metamour relationship, which is consistent with the idea of compersion. However, our exclusion of the ENC scale in the Polish version of the COMPERSe may suggest that mirroring of one’s partner’s feelings may not be primary mechanism behind compersion. It is noteworthy that in the original research (Flicker et al., 2021), the emotional contagion was not strongly related to compersion. Moreover, in our study we observed a relationship between compersion and cognitive empathy, which was also somewhat equivalent to compersion in open relationships compared to polyamory when it comes to affecting relationship satisfaction. (These issues will be detailed in the following paragraphs.). It is especially interesting because cognitive empathy can be exercised and developed (Teding van Berkhout & Malouff, 2016). Considering that—although it is possible that some people are more inclined to experiencing compersion—it may be assumed that compersion should be conceptualized more like a competence and capability, but not as a trait (or at least HPMR should be conceptualized in that way). This idea finds support in the literature, as some participants in Flicker et al.’s (2022) study reported that their “ability to experience compersion developed over time often as a result of purposeful cultivation” (p. 3038).

### Divergent and Convergent Validity

Generally, both divergent and convergent validity analyses indicated that relations between validity measures and the COMPERSe subscales were consistent with predictions, suggesting that the validity of the Polish version of the COMPERSe was fairly strong. We observed a negative relation between HPMR scale and anxiety, as well as positive correlations between HPMR and emotional intelligence, self-esteem, and relationship satisfaction. Relationship between SA and relationship satisfaction also matched our predictions. Jealousy, especially the Threat to Relationship Exclusivity and Aversion to Partner’s Autonomy subscales, was negatively related to the HMPR scale. The relationship between HPMR and Self-abuse/Envy was significant, but not very strong, and the Dependence was not related to HPMR at all. Meanwhile, we observed a rather weak negative relation with SA and Aversion to Partner’s Autonomy, and, surprisingly, weak but positive with SA and Dependence. Though most of these findings are consistent with previous research (Aumer et al., 2014; Duma, 2009; Flicker et al., 2021), the last relation mentioned requires some reflection. It may be that more dependent individuals eroticize the emotional pain of jealousy as a part of the compersive response (cf. Deri, 2015). Another possibility is that the observed relationship is in fact coincidental, as the reliability of Dependence was not that high (Cronbach’s Alpha in the CNM subsample = 0.69).

Similarly to the original research (Flicker et al., 2021), we observed positive relations between cognitive empathy and compersion. As we discussed in the previous paragraph, since cognitive empathy is a skill that can be trained (Teding van Berkhout & Malouff, 2016), we assume that compersion comes from practicing specific thinking, understanding other’s needs, and reconceptualizing the underlying monogamous way of thinking about intimate relationships (cf. Flicker et al., 2022).

Finally, an important matter is the relationship between compersion and attachment styles. As expected, anxious and avoidant styles were negatively related to HPMR, and secure style has positive relations to both HPMR and SA. The attachment theory is usually understood through monogamous lens, dyadic framework, and it states that insecurely attached people, especially avoidantly, are more prone to exhibit non-monogamous behaviors (cf. Katz & Katz, 2022). Balzarini and Muise (2020, p. 402) stated that “sexual and romantic exclusivity is often conflated with ideas of love among attachment theorists” (see, e.g., DeWall et al., 2011; Hazan et al., 2006; Hazan & Zeifman, 1999). That being said, research results indicate that polyamorists are usually securely attached (e.g., Moors et al., 2019; Morrison et al., 2013; cf. Katz & Katz, 2022), while highly avoidant individuals are rather more attracted to the idea of CNM itself, but do not necessarily engage in practicing CNM (Ka et al., 2022; Moors et al., 2015). It may be because practicing CNM requires characteristics of secure attachment style, such as constructive and open communication, high levels of honesty, trust, and intimacy (Balzarini et al., 2017, 2019, 2021; Katz & Katz, 2022; Mogilski et al., 2017; cf. Hazan & Shaver, 1987), which leads to greater sense of safety within relationships (Lessin et al., 2005). Polyamorous individuals may also be more comfortable with intimacy than monogamous individuals (Morrison et al., 2013). Taking all this into account, it seems reasonable that a higher level of compersion, which is an important factor among CNM individuals, especially polyamorous, is positively related to the secure attachment style, with which it shares characteristics.

Though relationships between both compersion scales and other measures in monogamous sample were almost nonexistent, there were some interesting correlations worth brief discussion. Attachment styles were not related to compersion except for the secure attachment style that was negatively related to SA. These results match the classic, unnuanced definition of secure attachment that more secure attached monogamous individuals strive to maintain close, intimate exclusivity with only one partner (cf. Balzarini & Muise, 2020; Katz & Katz, 2022).

Similar to the CNM sample, Threat to Relationship Exclusivity and Aversion to Partner’s Autonomy were negatively related to both of the COMPERSe scales. It means that regardless of monogamy or non-monogamy, positive emotions related to compersion are either perceived or felt in contrast to emotional pain of jealousy. However, we also observed a positive relation between SA and Self-abuse/Envy. Envy, more than jealousy, results from the hateful comparison of one's own characteristics and achievements with the characteristics and achievements of others, which is accompanied by the desire to match a given person (Zaleski & Hupka, 1991; cf. Smith et al., 1988). Thus, monogamists who feel more envious of a potential person having relationships or characteristics that one desires tend to feel more sexual excitement, arousal, and be more turned on while thinking of a possibility that their partner may have another partner. It is possible that some monogamous people may tend to eroticize envy (cf. Deri, 2015) or find some pleasure in envious pain, which is similar to some forms of kink, especially cucking. However, this may be too far-reaching interpretation and it requires more extensive research.

### Intergroup Differences

In a second major group of analyses, we tested the differences between CNM and monogamous participants. CNM participants experienced more compersion (both sexual and emotional) than monogamous participants, confirming the high validity of the COMPERSe. These results are not discussed in detail, as compersion relates directly to non-monogamous experiences and monogamous participants referred to their feelings regarding the possible situation of having metamour.

Other differences observed were those regarding jealousy. In every measured aspect of jealousy, monogamous people achieved higher results than CNM participants, which is consistent with both intuitive assumptions and some research results. For example, Aumer et al. (2014) observed that jealousy positively predicted relationship satisfaction only in monogamous relationships and that compersion had no effect on satisfaction. This is also consistent with the function of jealousy, which acts as a protection of [monogamous] relationship exclusivity (Zaleski & Hupka, 1991). Similarly, lower scores in jealousy among CNM participants may be a result of compersion mitigating the magnitude of jealousy that, in non-exclusive CNM relationships, does not contribute positively to relationship satisfaction (cf. Aumer et al., 2014; Flicker et al., 2021).

Another interesting difference was that CNM participants had higher cognitive empathy scores than monogamous participants. The fact that cognitive empathy can be trained (Teding van Berkhout & Malouff, 2016) suggests that CNM people may be more cognitive empathetic because of possible training obtained by practicing consensual non-monogamy itself. The reason for this is that CNM most probably requires taking into account perspective, feelings, and needs of every individual involved to establish a well-functioning CNM relationship.

CNM individuals also exhibited anxious attachment style traits less often than monogamous people, but the other two styles characteristics (secure and avoidant) were more or less on about the same level in both groups. It is interesting because some researchers speculated that anxious individuals may be likely to practice CNM to seek more affection and attention or engage in CNM “at their partner’s request” (Ka et al., 2022). Meanwhile, Katz and Katz (2022) regarded secure and anxious individuals to be more prone to engage in CNM. In our research anxious style was most closely and positively associated with different types of jealousy in both CNM and monogamous groups (0.27 < *r*s < 0.58, *p*s < 0.001) and was also most closely and negatively associated with compersion among CNM individuals. Thus, we speculate that anxious attachment characteristics are less manifested among CNM individuals than monogamous individuals because they are associated with negative emotions that make it difficult to engage in requiring more trustful style of thinking consensual non-monogamy.

The last difference observed, that the CNM individuals were generally more satisfied with their relationships than monogamous individuals, was somewhat surprising. To our knowledge, none of the studies systematically demonstrated this effect, although some research revealed that CNM people are highly satisfied with their relationships (Garner et al., 2019) and that they report higher security scores than established norms among monogamists (Moors et al., 2019). It may be because practicing CNM requires communication about outside dyadic relationships, which can improve the quality of dyadic relationships, helping them to be more satisfying and fulfilling (Katz & Katz, 2022). It is also possible that CNM participants were more satisfied with their relationship(s) because dating with other people being in non-traditional minority may support better understanding of each other by bringing out similarities (partners selection is more deliberate and less random).

Another part of the intergroup differences analyses was about different types of CNM. Unfortunately, only people in polyamorous and open relationships met the sample size conditions. Consistent with speculations, polyamorous individuals had higher levels of HPMR, but SA scores were similar between both groups. It is not surprising because compersive sexual arousal could be similarly high among each of these CNM groups, since neither assumes sexual exclusivity. However, only polyamory does not assume romantic exclusivity (Fern, 2020), hence a lower score in HPMR among participants in open relationships.

Additionally, participants in open relationships showed a higher aversion to partner’s autonomy than polyamorous participants. It may be due to open relationships tend to be focused more on sexual non-exclusivity, keeping dyadic emotional involvement as primary, which shares similarities with monogamy (see: Fern, 2020). Since aversion to partner’s autonomy refers to the degree of control that one wants to have over one’s partner (Zaleski & Hupka, 1991), it is reasonable that people in open relationships may feel more jealous.

Lastly, secure attachment characteristics were more exhibited by polyamorous individuals than individuals in open relationships. It could be because secure attachment allows for more constructive communication, healthy emotional expression, and greater sense of safety—characteristics that can help one deal with multiple romantic relationships, which polyamory covers. Contrary, secure attachment traits may not be such a necessity in in open relationships, where outside dyadic relationships are primarily sexual and usually do not involve romantic intimacy.

### Mediation Models

In the third major group of analyses, we discovered that the relationship between compersion (especially HPMR) and relationship satisfaction was not direct and was mediated by aversion to partner’s autonomy, one of the aspects of jealousy. This result is consistent with existing literature, in which it is speculated that compersion may mitigate the magnitude of jealousy, consequently contributing to increased relationship satisfaction (Flicker et al., 2021).

Although the relationship described was not surprising itself, it is not obvious why it was aversion to partner’s autonomy which mediated the effect, but not other aspects of jealousy, namely threat to relationship exclusivity. The role of the latter would appear even potentially better due to its relatively high negative correlation with compersion (HPMR) and the fact that in CNM relationships the lack of partner’s exclusivity is a fundamental property. However, according to definitions of both aspects of jealousy (Zaleski & Hupka, 1991), threat to relationship exclusivity refers to cognitive and physiological reactions when someone is excluded from their partner's activity or when the partner directs attention to other people. Meanwhile, aversion to partner’s autonomy refers to the degree of control that the jealous person wants to have over the partner. Therefore, a mere threat to relationship exclusivity may not necessarily involve taking any action or visible behavior directed at a partner, such as participation in partner’s activities. What is important, such jealous participation is not a direct purpose—it is not about participation itself, but about the benefits of being in control. It allows for monitoring one’s partner’s behavior to be ready to react if any interaction between one’s partner and one’s metamour causes one to feel insecure—such characteristics are associated with aversion to partner’s autonomy. Therefore, we think it is reasonable that compersion can mitigate exactly that aspect of jealousy.

It is noteworthy that this observation (that compersion may influence relationship satisfaction by decreasing the tendency to take control over partner and partner activities rather than reduce cognitive and physiological reactions) adds some information about the characteristics of CNM. It is possible that a person who is highly controlling the behavior of their partner may have more problems in establishing healthy CNM relationships without [practicing] compersion. Such a person may find it not difficult to deal with negative emotions that accompany a situation when their partner is on a date with their metamour, but at the same time, such a person may keep an eye on their partner’s arrival time or may demand the exact details of the meeting to be told. A reverse situation, when one feels jealous that one’s partner is doing something without one, but one does not want to control one’s partner, may be more typical for “don’t ask, don’t tell” type of relationships, but not for open or polyamorous relationships.

Next, we discovered that our mediation model can be enriched with another variable, cognitive empathy. It predicted relationship satisfaction in two ways: (1) by facilitating compersion, which lowered aversion to partner’s autonomy, resulting in higher relationship satisfaction, and (2) by directly decreasing jealousy. As we mentioned in previous paragraphs, the observation that cognitive empathy predicted compersion, suggests that compersion may be conceptualized as a competence and capability involving processes that can be consciously acquired. It is because cognitive empathy, but not emotional empathy, can be learned during training (Teding van Berkhout & Malouff, 2016). Awareness of this possibility may help people to consciously work on their non-monogamous relationships, as some may unwittingly expect compersion to just “appear spontaneously or naturally.” This expectation, without practicing compersion (rather than focusing on feeling of compersion), may lead to anxiety and disappointment, and further to not mitigating potential jealousy that can have detrimental influence on relationships.

Further revision of the mediation model revealed important differences between people in polyamorous and open relationships, as well as differences between these groups in relation to the general model. For polyamory, compersion directly predicted relationship satisfaction. In addition, compersion was not predicted by cognitive empathy. On the other hand, for open relationships, it was cognitive empathy, but not compersion, that directly predicted relationship satisfaction. Though compersion had no effect on relationship satisfaction, it was predicted by cognitive empathy. Additionally, in both open and polyamorous relationships compersion decreased jealousy (and so did cognitive empathy, but only in open relationships), but at the same time jealousy had no effect on relationship satisfaction.

The reasons for such major differences may be explained as a result of differences in the characteristics of polyamory and open relationships. It was suggested that compersion refers primarily to polyamorous communities (Deri, 2015; Ferrer, 2008), and is discussed mostly in online polyamorous communities, blogs, and popular literature that refer to polyamory (e.g., Anapol, 1998; Ritchie & Barker, 2006; Sheff, 2013; Taormino, 2008; cf. Flicker et al., 2021). This term was also coined by members of the Kerista polyfidelitous commune (Deri, 2015; Pines & Aronson, 1981). Thus, it can be expected that compersion (especially emotional compersion measured by HPMR) may be far more important factor in the case of polyamorous, but not open relationships.

At the same time, open relationships put less emphasis on engaging in multiple romantic relationships, instead concentrating more on outside dyadic sexual relationships (e.g., Conley et al., 2017; Fern, 2020; Matsick et al., 2014). Therefore, to establish a satisfying open relationship compersion may not be required to be in such magnitude as in polyamory or may not be required at all, because it may not apply to the mere idea of open relationships. Instead, to function better, open relationships may need the equivalent of compersion—following our analysis, it could be cognitive empathy. It may allow to understand one’s partner’s needs, allowing to accept the fact that one’s partner can meet their sexual needs with other people, which does not have to be accompanied by pleasure, joy and satisfaction resulting from the awareness of it, which refers to compersion (Mogilski et al., 2019). At the same time, greater cognitive empathy can increase compersion as a by-product that has no major impact on relationship satisfaction but may help cognitive empathy reduce jealousy.

### Limitations, Implications, and Future Research

Though our study contributes to the growing number of research concerning consensual non-monogamy and compersion, it has several limitations we would like to discuss. Some of them are associated with the limitations of the research by Flicker et al. (2021), and we decided to refer to the most important as well.

The most important issue is that the Polish adaptation of the COMPERSe contains items and instruction requiring subjects to choose only one of their partners. As Flicker et al. (2021) noticed, one may select the partner with whom the relationship is associated with the greatest compersion, making the results biased. For now, we could not perform research that would verify whether the psychometric values of the questionnaire would show comparable results when completed regarding other partners, because our first step was to adapt the COMPERSe to the same conditions that were in the original study. However, exploring that issue is one of the most necessary future steps to make COMPERSe a more universal measure.

Second, but strongly related to the first issue, the COMPERSe in both English and Polish versions is designed to assess one’s feelings about a specific partner–metamour relationship (and additionally, in the case of English version, to more abstract potentially new intimate connections). Therefore, it is more suited to measure compersion conceptualized as a state, but not as a trait. Flicker et al. (2021) suggested that the assessment of a trait compersion would be possible if one evaluates their feelings toward more than one of their partner–metamour dyad and then the scores will be averaged. However, it can be an issue of anchoring bias heuristic if one would complete the questionnaire multiple times regarding multiple cases, adjusting the evaluation of one case to the previous (or first) case evaluation. That would result in excessively similar assessments, making the testing less reliable. It is possible that to assess a trait compersion it is necessary to create a new questionnaire with new items, based on these in the COMPERSe, but formulated differently.

Considering implications of our research, the most important one seems to be simply the adaptation of the first and, to date, the only fully standardized quantitative scale designed to measure compersion to the Polish-speaking population. In nearly nonexistent Polish research on non-monogamy and compersion, it seems that only unvalidated measures (Duma, 2009) were used (Frycz, 2019), making difficult to compare the results of different studies. Therefore, we hope that the COMPERSe, a standardized scale, will be used in future research, and that its existence will facilitate the emergence of new studies. 

As noted by Flicker et al. (2021), research on compersion has potential implications on relationship therapy. Although experiencing compersion is often identified as a goal among polyamorous people, it is not realized in satisfying way by many of them (Deri, 2015; Ferrer, 2008). In addition to psychometric adaptation, our study also presented several models showing relations between compersion, jealousy, empathy, and relationship satisfaction. Knowing these relations, therapists may find it helpful when they are working with people who find it difficult to manage jealousy or to have higher satisfaction with their non-monogamous relationships. Furthermore, understanding the relationships between these factors may contribute to the development of new training methods strategies to increase relationship satisfaction. Several studies indicate that cognitive empathy can be learned during training (cf. Teding van Berkhout & Malouff, 2016); thus, increasing cognitive empathy may also lead to increasing compersion and further to decreasing jealousy, finally resulting in increased relationship satisfaction. Also, considering the differences between polyamorous people and people in open relationships, it would be valuable to additionally develop training directly increasing compersion. Such training, if effective, would also give arguments for the idea to conceptualize compersion as a competence that can be acquired. Anyway, future research should investigate further the issue of differences between different forms of non-monogamy concerning the relations between compersion, cognitive empathy and relationship satisfaction.

## Data Availability

The materials for all experiments are available in the Online Supplement; the data and the supplement are available at OSF data repository: https://osf.io/cz3sd; the study was preregistered: https://osf.io/6nqk5.
